# Analyzing and computing humans by means of the brain using Brain-Computer Interfaces - understanding the user – previous evidence, self-relevance and the user’s self-concept as potential superordinate human factors of relevance

**DOI:** 10.3389/fnhum.2023.1286895

**Published:** 2024-02-16

**Authors:** Cornelia Herbert

**Affiliations:** Department of Applied Emotion and Motivation Psychology, Institute of Psychology and Education, Ulm University, Ulm, Germany

**Keywords:** brain–computer interfaces, human factors, self-relevance, self-concept, user traits and states, SMR-BCI, P300-BCI, BCI performance

## Abstract

Brain–computer interfaces (BCIs) are well-known instances of how technology can convert a user’s brain activity taken from non-invasive electroencephalography (EEG) into computer commands for the purpose of computer-assisted communication and interaction. However, not all users are attaining the accuracy required to use a BCI consistently, despite advancements in technology. Accordingly, previous research suggests that human factors could be responsible for the variance in BCI performance among users. Therefore, the user’s internal mental states and traits including motivation, affect or cognition, personality traits, or the user’s satisfaction, beliefs or trust in the technology have been investigated. Going a step further, this manuscript aims to discuss which human factors could be potential superordinate factors that influence BCI performance, implicitly, explicitly as well as inter- and intraindividually. Based on the results of previous studies that used comparable protocols to examine the motivational, affective, cognitive state or personality traits of healthy and vulnerable EEG-BCI users within and across well-investigated BCIs (P300-BCIs or SMR-BCIs, respectively), it is proposed that the self-relevance of tasks and stimuli and the user’s self-concept provide a huge potential for BCI applications. As potential key human factors self-relevance and the user’s self-concept (self-referential knowledge and beliefs about one’s self) guide information processing and modulate the user’s motivation, attention, or feelings of ownership, agency, and autonomy. Changes in the self-relevance of tasks and stimuli as well as self-referential processing related to one’s self (self-concept) trigger changes in neurophysiological activity in specific brain networks relevant to BCI. Accordingly, concrete examples will be provided to discuss how past and future research could incorporate self-relevance and the user’s self-concept in the BCI setting – including paradigms, user instructions, and training sessions.

## 1 Introduction

Brain–computer interfaces (BCIs) convert brain activity into adaptive and assistive computer commands. This makes it possible for BCI users to interact and communicate with their environment non-invasively solely by changes in neural activity detected for example by electroencephalographic recordings (EEG). Conventional use cases of BCI applications are EEG-based control of external devices such as a robotic hand, limb or foot. The ultimate goal of using BCIs in this context is to give patients, who are unable to communicate directly through other means, such as patients with motor impairments or neurological impairments, a clinically supportive aid for communication or interaction (for an overview see e.g., Kleih et al., 2011a; Luauté et al., 2015). Progress in BCI technology has made it possible to utilize BCIs for a wide range of clinical user groups. These user groups may include elderly patients or individuals with mental health conditions suffering from stress, depression, cognitive decline, or cognitive symptoms such attention deficit hyperactivity, to name a few examples (e.g., Balcombe and de Leo, 2022). Moreover, a considerable body of BCI research has spotted the healthy user. Use cases of BCIs among healthy users can include the attempt to control the driver’s fatigue during automated driving (e.g., Zhang et al., 2020) or the monitoring and support of the user’s mental state in challenging occupational settings (e.g., a pilot’s attention during a challenging flight maneuver; Dehais et al., 2022). The use of BCIs in occupational settings and even more so simply for fun, entertainment, or leisure activities is growing (Nijholt et al., 2022). Use cases include brain-controlled gaming and sports (e.g., Randolph, 2012; Diya et al., 2019) for example in virtual reality augmented digital settings. According to recent research, expanding the applications of BCIs to recreational activities or as a tool for the workplace could enhance the scientific knowledge about training-induced or learning-induced neuroplasticity in a variety of users (for a comprehensive overview or recent research on occupational neuroplasticity, refer to e.g., Wang et al., 2017; Wu et al., 2020). Therefore, understanding the BCI user has become an important topic in BCI research.

## 2 The human user in BCI research

A body of studies has shown that despite technical advances in the field, variance in BCI performance is owing to the user. According to previous estimations, about 20% up to 40% of BCI users may not achieve a BCI performance with the necessary accuracy to benefit from an BCI application (for an overview see e.g., Edlinger et al., 2015; for recent estimations and discussions concerning motor imagery based BCI, e.g., see Zhang et al., 2020).

“BCI inefficiency” or “BCI illiteracy” (Allison and Neuper, 2010) has been found among healthy users, as a mentally and behaviorally fully responsive user group of BCIs or among patients, who are mentally handicapped or behaviorally non-responsive (e.g., Guger et al., 2003; Allison and Neuper, 2010; Graimann et al., 2010). This has encouraged discussions about the relevance of research on human factors not only in the domain of human–computer interaction (HCI) in general, but in the domain of BCI research, its deployment and application as well (for an overview see for example, Botte-Lecocq et al., 2014; Kleih and Kübler, 2015). Furthermore, the discussion on the role of the BCI user has promoted theoretical suggestions of how to integrate human factors into BCI technology by means of a user-centered approach (for an overview see e.g., Kübler et al., 2015, 2020; Alonso-Valerdi and Mercado-Garcıa, 2019; or for motor imagery based BCIs, e.g., Lyu et al., 2023). Additionally, guidelines of BCI training protocols have been suggested to incorporate human factors into the BCI settings (Mason and Birch, 2003; Botte-Lecocq et al., 2014; Jeunet et al., 2018).

### 2.1 Human factors related to BCI engineering and ergonomics

In conjunction with these theoretical efforts, several studies have specifically looked into human factors that, when considering the BCI’s design features, may have an impact on the device’s usability and practicability. To this end, the user’s satisfaction with the BCI system (e.g., Zickler et al., 2009; for a discussion see Kübler, 2020), or the user’s effectiveness of BCI use or the user’s acceptability have been investigated among healthy users or patients. In addition, the user’s previous experience with technology (Al-Taleb et al., 2019; Voinea et al., 2019; Leeuwis et al., 2021; for a discussion, see Kübler et al., 2014) or the mental workload and fatigue imposed by the BCI task and training routines have been examined to this end (e.g., Käthner et al., 2014). Furthermore, some studies investigated if sociodemographic variables like the experimenter’s or user’s age and gender affect the user’s BCI experience. A number of studies found that both factors (gender and age) can modulate the experience of BCI users (Randolph, 2012; Zich et al., 2017; Wood and Kober, 2018; Pillette et al., 2021). In conclusion, the results of these studies suggest that specific human factors are either strongly restricting or enhancing human factors for BCI engineering and ergonomics. For recent reviews on the role of human factors for BCI design engineering, see e.g., Saha et al. (2021); for P300-spellers e.g., Powers et al. (2015); for SMR-BCIs, see e.g., Lyu et al. (2023).

### 2.2 Psychological human factors: user traits, states, and BCI performance

Furthermore, a significant body of research has investigated how human factors, specifically psychological factors related to the user’s mental traits or state influence BCI performance. Psychological human factors that relate to the BCI user’s mental states or traits are factors such as the user’s personality (trait) or current motivation, mood, emotion, or affect, or the user’s cognitive skills and aptitudes such as the user’s ability to pay attention, the user’s learning capacities, intelligence, self-regulation or cognitive control strategies. Remarkably, these user traits and states might affect BCI performance explicitly or implicitly (e.g., hidden from direct observation) by modulating the different BCI’s outcome measures of interest on a behavioral, subjective or neural level (e.g., accuracy measures or brain signals used for BCI classification). Moreover, not all psychological user traits and states of interest are directly accessible for the users themselves via introspection or self-report. Thus, the assessment of such factors benefits from sophisticated examination by measures of experimental manipulation or psychological testing.

## 3 Challenges of previous BCI human factor research and aims of the present study

Essentially, the key to increasing BCI literacy and decreasing BCI inefficiency across and beyond the individual BCI user, is knowing which of the psychological user’s traits and states are influencing BCI performance, when, how, and in whom across the BCI types and tasks, or technical solutions.

**FIGURE 1:**
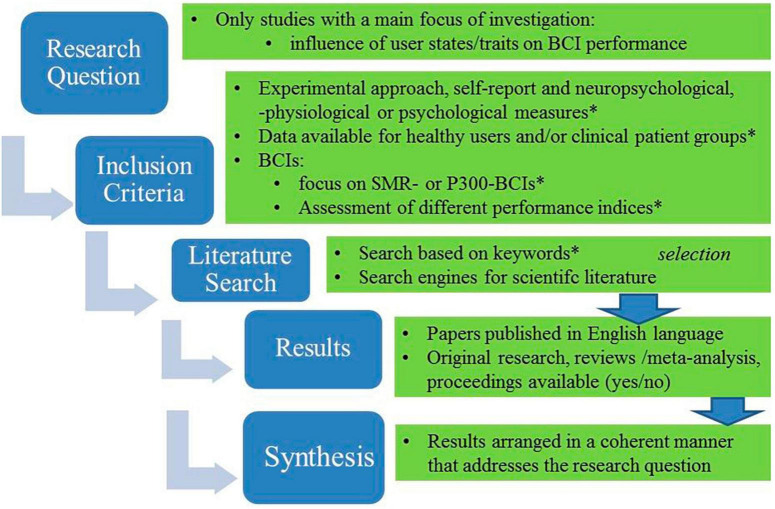
Overview of the selection procedure of the previous studies summarized in the section “4 Psychological human factors and their impact on BCI performance: evidence from previous studies using P300-BCI or SMR-BCI among healthy and disabled user groups” that investigated user traits and states. Asterisk indicates the key words for the literature search.

Up to now and as summarized above, several studies already examined human factors in the context of BCIs. For human psychological factors that influence neurofeedback learning outcomes, see for example, Kadosh and Staunton (2019). For recent overviews on SMR-BCIs and internal factors, see for example, Horowitz et al. (2021), or the previous reviews by Ahn and Jun (2015), Roc et al. (2021), or Wierzgawa et al. (2018). Despite these previous attempts, so far, it is difficult to draw definitive conclusions about the overall influence of user traits and states specifically on BCI performance across BCIs and user groups. Such overviews are currently still rare in the literature, probably because of variations among the different studies in study protocols and methodology. From a methodological point of view, however, a number of the existing previous studies are available whose main goal was to investigate psychological human factors, specifically the impact of user characteristics or states on BCI performance (see Figure 1) and that used similar study protocols. For example, these studies either experimentally manipulated the user’s traits and states, or measured the user’s traits and states via psychological test batteries or via standardized self-report. As a result, the evaluation techniques were carefully controlled and comparable among studies. In addition, among these studies, a number of studies explored the user’s traits and states on BCI performance among healthy users or disabled patient groups with similar protocols. The BCI systems comprised well-established non-invasive EEG-BCIs that were either based on motor imagery as a task to elicit sensorimotor rhythms (in short: SMR-BCIs or MI-BCIs) or on P300 modulation elicited by the voluntary attention of the user (e.g., P300-BCI spellers), for further details, see section “4 Psychological human factors and their impact on BCI performance: evidence from previous studies using P300-BCI or SMR-BCI among healthy and disabled user groups.”

As a result, these studies could allow a valid comparison of the effects of user traits and states on BCI performance across two different but very frequently used BCIs, SMR-BCI and P300-BCI, respectively. Moreover, their findings might allow a first cross-sectional evaluation of how user traits and states influence BCI performance across user groups and across the two different but well-established BCI systems of SMR-BCI and P300-BCI. Most importantly for future aims, recommendations concerning superordinate or key human factors that could influence BCI performance beyond tasks and BCI applications can be made based on a joint analysis of these studies’ findings.

**FIGURE 2:**
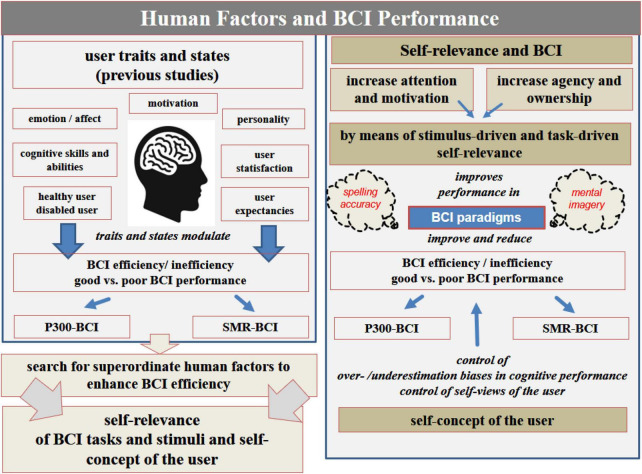
**(Left column)** Overview of the user traits and states examined in previous studies using SMR-BCI or P300-BCI in samples of healthy users and patients (for a detailed summary see Table 1 and section “4 Psychological human factors and their impact on BCI performance: evidence from previous studies using P300-BCI or SMR-BCI among healthy and disabled user groups”). **(Right column)** Self-relevance as a key human factor, its potential influence on BCI performance and its implementation in the BCI design. For a detailed discussion, see section “Self-relevance and the user’s self-concept as potential superordinate human factors in the BCI setting” and subsections in the text and Figures 3, 4.

**FIGURE 3:**
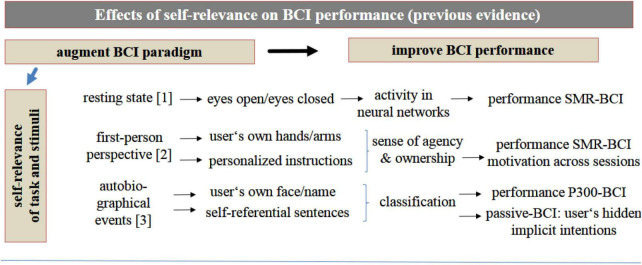
Self-relevance and BCI performance as illustrated by different examples discussed in the text in section “Self-relevance and the user’s self-concept as potential superordinate human factors in the BCI setting” and subsections. The number in brackets refer to the studies discussed in section “Self-relevance and the user’s self-concept as potential superordinate human factors in the BCI setting.” [1] Resting state (e.g., Blankertz et al., 2010), [2] first person perspective or own body parts (e.g., Neuper et al., 2005; Škola and Liarokapis, 2018; Ziadeh et al., 2021) and [3] autobiographical events (e.g., Dong et al., 2015; Placidi et al., 2018).

**FIGURE 4:**
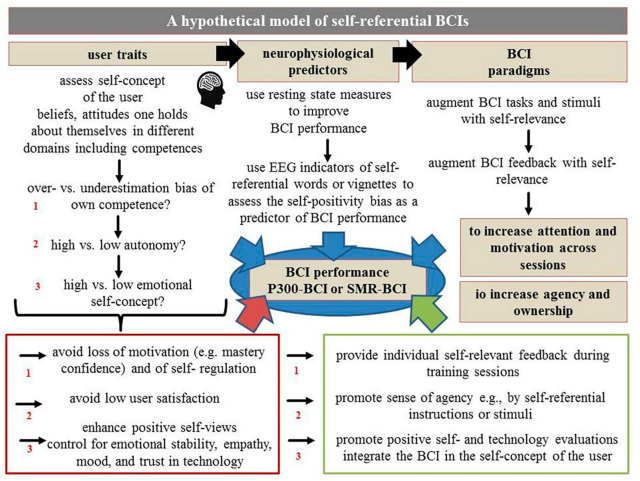
Hypothetical model that illustrates the possible relationship between the self-concept of the user, the self-relevance of tasks and stimuli, BCI performance, and the previously examined user traits and states. For a detailed discussion, see section “Self-relevance and the user’s self-concept as potential superordinate human factors in the BCI setting.”

In line with this, the following sections of this manuscript aim to provide a brief overview, review and discussion of the findings of these studies that investigated psychological human factors. Selection of the studies of interest was based on a literature search according to the criteria mentioned above and as illustrated in detail in Figure 1. Based on the summary of the research findings from these studies (see also Table 1 and Figure 2), the following sections will propose that, in accordance with the above recommendations, self-relevance, along with the user’s self-concept, may be potential superordinate human factors that affect BCI performance both within and across the well-investigated BCIs (P300-BCIs or SMR-BCIs, respectively). Consequently, concrete examples and a hypothetical model discussed in the following sections (for a graphical summary see Figures 3, 4) will demonstrate that self-relevance and the user’s self-concept should be thoroughly examined in subsequent BCI studies. These studies might also include BCIs other than P300- or SMR-BCIs.

**TABLE 1:** This table provides an overview of the studies discussed in the subsections of section “4 Psychological human factors and their impact on BCI performance: evidence from previous studies using P300-BCIs or SMR-BCIs among healthy and disabled user groups.”

Human factors (HC) assessment method – HC	User population	BCI	Results	References
**Motivation** **(state)** via experimental manipulation using monetary reward during the spelling sessions (extrinsic motivation, state) and self-rated current motivation assessed with the visual analog scale (VAS) and the standardized Questionnaire for Current Motivation (QCM-BCI). Mood (state) was assessed with a subscale of a quality of life questionnaire and with the VAS.	33 healthy students (4 male, 29 female), all naïve to BCI training.	P300-BCI	Relationship between P300 amplitude and self-rated motivation (VAS). Faster BCI-based communication among highly motivated participants than among less motivated participants. No differences in CRR as a function of monetary reward. Significant decrease of the P300 amplitude for the groups with no or low reward and no decrease in P300 amplitude in the group with high reward (between calibration and copy-spelling). Self-reported motivation was not related to reward.	Kleih et al., 2010
**Motivation (state)** via self-report: standardized Questionnaire for Current Motivation (QCM-BCI) that assesses current achievement motivation related to BCI use and the learning process including four motivational components “mastery confidence,” “incompetence fear,” “challenge,” and “interest.” Rating of current motivation (state) using the VAS.	90 participants, 13 diagnosed with amyotrophic lateral sclerosis (ALS), mean age: 29.29 years.	P300-BCI	Significant positive correlations between BCI performance (accuracy) and motivation as measures by the VAS and the QCM-BCI (incompetence fear). Effects not reported separately for healthy vs. patient users.	Kleih et al., 2011b
**Motivation (state/trait)** via self-report: custom-made questionnaire for separation of highly intrinsically vs. extrinsically motivated participants from less intrinsically vs. extrinsically motivated participants (state/trait) AND standardized Questionnaire for Current Motivation (QCM-BCI) including the four motivational components “mastery confidence,” “incompetence fear,” “challenge,” and “interest.” In addition, ratings of current motivation (state, VAS). **Affect and personality** **(trait)** via standardized questionnaires including four subscales “perspective taking,” “fantasy” (FS), “empathic concern” (EC), and “personal distress” (PD) and Interpersonal Reactivity Index (IRI). **Empathy (trait)** via questionnaire using the subscale “agreeableness.” **Cognition** via test battery including attention allocation and memory. **Mood** including positive and negative affect (trait) and depressive symptoms (last 2 weeks, via depression scale) as further indicator of mood changes. Manipulation check: interest in BCI, cognitive effort/exhaust post measurement.	21 healthy students, 14 female, mean age: 23.35 years, naive to BCI.	P300-BCI	Higher P300 amplitude in participants with low empathy. No effects of motivation nor of any of the other measures.	Kleih and Kübler, 2013
**Motivation** via experimental manipulation using monetary reward during the motor training sessions (extrinsic motivation, state) and self-rated motivation via VAS AND standardized Questionnaire for Current Motivation (QCM-BCI) including the four motivational components “mastery confidence,” “incompetence fear,” “challenge,” and “interest.” **Emotion (state)** induction via experimental manipulation inducing negative and neutral emotional states (video clips and pieces of music). Mood ratings of valence and arousal (Self-Assessment Scale). **Mood (trait)** via quality of life scales and self-reported depressive symptoms.	42 healthy volunteers, naïve to BCI training, mean age: 24.22 years.	SMR-BCI	Increased task-related brain activity in extrinsically motivated (rewarded) as compared to non-motivated (unrewarded) participants but no clear effect of emotional state manipulation on BCI performance.	Kleih-Dahms et al., 2021
**Mood** via quality of life scales and self-reported depressive symptoms AND standardized Questionnaire for Current Motivation (QCM-BCI) including the four motivational components “mastery confidence,” “incompetence fear,” “challenge,” and “interest.”	16 healthy adult participants naive to BCI (students, 6 men, 10 women), divided into two groups with average mean age: 26.75 or 24.23 years.	SMR-BCI	Main effect of mood, mastery confidence and fear of incompetence on BCI performance (visual feedback group). In the auditory feedback group, main effect of incompetence fear only: higher scores of incompetence fear were related with better performance.	Nijboer et al., 2008
**Psychological wellbeing** and changes in mood via self-report, measured via quality of life (state/trait) standardized questionnaire. **Mood** including depressive symptoms measured via depression scale. **Motivation (state)** via self-report by standardized Questionnaire for Current Motivation related to BCI use (QCM-BCI) including the four motivational components “mastery confidence,” “incompetence fear,” “challenge,” and “interest.”	6 ALS patients, age: 39–67 years.	SMR-BCI and P300-BCI	Relationship between mood, motivation, and number of BCI sessions. Relationship between BCI performance and motivational factors (BCI) – challenge, mastery confidence and incompetence fear in half of the participants but no relationship between mood and BCI performance in any of the participants.	Nijboer et al., 2010
**Motivation (state)** via self-report using the Questionnaire for Current Motivation related to BCI use (QCM-BCI) including the four motivational components “mastery confidence,” “incompetence fear,” “challenge,” and “interest.” Additionally, ratings of current motivation (state) via VAS. **Subjective Workload** **(state)** via self-report using a standardized questionnaire. **Mood** including depressive symptoms assessed via depression scale. **Satisfaction own performance (state)** assessed via VAS. **Cognition:** Attention test.	16 university students (8 female), mean age: 23.88 years.	P300-BCI	Relationship between current motivation (subscale “interest” and VAS rating) and BCI performance or P300 amplitude (VAS only). No other effects (mood, satisfaction, cognition) found or reported.	Baykara et al., 2016
**Physical, mental, and emotional states** via self-report using rating scales (Likert 1–5) for calmness, interest, concentration level, physical state, mental state, fatigue, and easiness of motor imagery and accuracy prediction (pre and post task).	52 healthy subjects (26 male, 26 female), mean age: 24.8 years.	SMR-BCI	No significant relationships between physical and mental states and BCI performance. Relationship between self-reported accuracy prediction and actual BCI performance.	Ahn et al., 2018
**Psychological trait and state markers** via electronic test battery and self-report measures including **Mental state:** self-reported depressive symptoms (state) and screening of psychopathological symptoms. **Current Mood (state)** via self-report. **Personality traits:** “empathy,” “emotional stability,” “extraversion, “conscientiousness,” “openness to experience,” and “agreeableness.” **Cognition** via electronic tests (e.g., sensorimotor coordination, attention and concentration, non-verbal intelligence (trait) and logical reasoning, verbal learning). **Self-control** via self-report scales (locus of control). **Motivation (state)** via standardized Questionnaire for Current Motivation related to BCI use (QCM-BCI) including the four motivational components “mastery confidence,” “incompetence fear,” “challenge,” and “interest.” **Attitudes towards work (trait)** via personality test including “performance motivation.”	83 healthy BCI novices (39 men), mean age: 29.5 years.	SMR-BCI	Relationship between BCI inefficiency and sensorimotor coordination, and between SMR-BCI performance and performance motivation/concentration. No other significant effects on BCI performance.	Hammer et al., 2012
**Full assessment of psychological trait and state markers**, same as in Hammer et al. (2012) plus additionally including: **Anxiety (state and trait)** via self-report/questionnaire. **General intelligence (trait)** via psychological tests. **Cognition** via cognitive tests comprising mental rotation, visuo-spatial short-term and working memory abilities, motor abilities, visual retention, and learning styles.	33 healthy participants (18 female, 14 male), mean age: 24.2 years.	SMR-BCI	Relationship between BCI performance and visuo-motor control ability and “attentional impulsivity” (personality trait).	Hammer et al., 2014
**Full assessment of psychological trait and state markers**, same as in Hammer et al. (2012, 2014).	40 healthy BCI novices (21 male, 19 female), mean age: 25.8 years, 92% students.	P300-BCI	Relationship between the personality factor “emotional stability” and visual or auditory P300-BCI performance. Relationship between non-verbal learning (ability to learn) and visual P300-BCI performance.	Hammer et al., 2018
**Psychological trait and state markers** including: **Anxiety (state and trait)**. **General intelligence (trait)** via psychological test. **Personality traits** including 16 primary factors of personality (warmth, reasoning, emotional stability, dominance, liveliness, rule-consciousness, social boldness, sensitivity, vigilance, abstractness, privateness, apprehension, openness to change, self-reliance, perfectionism, and tension) as well as five global factors of personality: extraversion, anxiety/neuroticism, tough mindedness, independence, and self-control. **Cognition** via test battery comprising mental rotation, visuo-spatial short-term and working memory abilities, motor proficiency, visual retention, and learning styles.	18 BCI-naive participants (9 female), mean age: 21.5 years.	SMR-BCI	Relationship between mental rotation and BCI performance. No other significant effects.	Jeunet et al., 2015
**Psychological factors (traits and states)** including: **Motivation (state)** via Questionnaire for Current Motivation related to BCI use (QCM-BCI) including the four motivational components “mastery confidence,” “incompetence fear,” “challenge,” and “interest” AND rating of current motivation (VAS). **Fatigue** via standardized scale. **Cognition and neurological and behavioral Functions** via test battery (working memory, cognitive flexibility). **General intelligence** via psychological test.	34 healthy participants.	P300-BCI	Working memory and general intelligence as significant predictors of BCI performance.	Sprague et al., 2016
**Full assessment of psychological traits and states** including: **Motivation (state)** via Questionnaire for Current Motivation related to BCI use (QCM-BCI) including the four motivational components “mastery confidence,” “incompetence fear,” “challenge,” and “interest.” **Personality traits** via standardized questionnaire (five factors: extraversion, autonomy, orderliness, emotional stability, and mildness). **Cognition** via psychological tests and questionnaires including visuo-spatial memory and spatial mental rotation and vividness of visual imagery (trait/state) as well as affinity to technology.	55 healthy and novice BCI-users (36 female, 21 male), mean age: 20.71 years.	SMR-BCI	Relationship between BCI performance and vividness of visual imagery and the personality factors: “orderliness” and “autonomy”.	Leeuwis et al., 2021
**Neurophysiological predictors** as external predictors of BCI performance and psychological **traits** (general intelligence/aptitude via questionnaire) for group selection.	20 healthy participants (7 female, 13 male) taken from Blankertz et al.’s (2010) sample, mean age: 24.5 years.	**SMR-BCI**	Differences in the activation of the supplementary motor areas (SMA) related to motor imagery and motor observation in high aptitude users.	Halder et al., 2011
**Neurophysiological predictor** of BCI performance (2 min EEG resting state, relax with eyes open) including control of self-reported fatigue.	80 participants, BCI-novices (41 female), mean age: 29.9 years.	SMR-BCI	Relationship between BCI performance and neurophysiological resting state predictor.	Blankertz et al., 2010
**Cognitive intervention:** visuomotor coordination training or progressive muscle relaxation. **Neurophysiological predictor** of BCI performance (same as in Blankertz et al., 2010).	154 naïve participants (99 female), mean age: 24.7 years.	SMR-BCI	No effects of either of the two cognitive interventions on BCI performance.	Botrel et al., 2017
**Psychological states including** **Motivation (state)** via standardized rating (VAS). **Relaxation (state)** via standardized rating (VAS). **Mood (state)** via standardized rating (VAS). **Mindfulness (trait)** via standardized scale and questionnaire. **Self-regulation (trait)** as the individual tendency to continue an action even if motivation and attention diminish, measured via standardized questionnaire. **Self-efficiency (trait):** cope with unexpected events via standardized scale. **Control beliefs – technology:** interaction with common technological devices at home or at work via standardized questionnaire. **Neurophysiological resting state predictor** of BCI performance (similar to Blankertz et al., 2010).	39 healthy people.	SMR-BCI	Relationship between BCI accuracy and the neurophysiological SMR predictor and state mindfulness. No relationships with psychological predictors.	Botrel and Kübler, 2019
**Mindfulness and meditation (trait)** via standardized questionnaires. **Neurophysiological differences (resting state)**.	16 healthy individuals with a history of meditation practice but no BCI experience, mean age: 38.5 years, 19 healthy individuals, mean age: 25.6 years, no meditation experience and no BCI experience.	SMR-BCI	Better task performance, fewer BCI inefficiency and higher resting SMR predictors in meditators compared to the control group.	Jiang et al., 2021
**Personality traits** via personality questionnaire assessing neuroticism (emotional stability) and extraversion/introversion. **Mindfulness training (state)** and motor exercise before or after the BCI sessions.	7 healthy subjects, mean age: 21–35 years.	SMR-BCI	Learning to control the BCI was modulated by neuroticism. Accuracy of the classification during motor imagery differed between subjects scoring high or low on the neuroticism scale before and after training. Motor training improved classification accuracy and mindfulness and motor training were modulated by neuroticism.	Bobrova et al., 2018
**User traits including personality traits** using different standardized questionnaires to assess. **Learning styles** (visual/verbal, active/reflective, sensitive/intuitive, or sequential/global). **16 personality factors** including warmth, reasoning, emotional stability, dominance, liveliness, rule consciousness, social boldness, sensitivity, vigilance, abstractness, privateness, apprehension, openness to change, self-reliance, perfectionism and tension. Global personality factors including extraversion, anxiety, tough-mindedness, independence, and self-control. **Cognition:** mental rotation test.	42 participants selected from three different studies/experiments that used the same MI-/SMR-BCI paradigm.	SMR-BCI	User traits including mental rotation and the personality factors of self-reliance and tension predicted BCI performances but not reliably across the experiments.	Benaroch et al., 2019

Human factors (trait or state) investigated in the studies are highlighted in bold letters. The overview considers the following major aspects: category of the human factor (user trait, user state), the assessment method (e.g., experimental, self-report, or test battery), the type of BCI used (P300-BCI or SMR-BCI), the user population (sample size, healthy sample or patient group), the main finding(s), and the study reference. The mean age of the study sample is reported as far as it is available. The study sample reported refers to the original sample size reported in the studies. Of note, the studies are not listed in chronological order of their publication (year of publication) but according to the order in which they are discussed in the text (see section “4 Psychological human factors and their impact on BCI performance: evidence from previous studies using P300-BCI or SMR-BCI among healthy and disabled user groups” and subsections).

## 4 Psychological human factors and their impact on BCI performance: evidence from previous studies using P300-BCI or SMR-BCI among healthy and disabled user groups

### 4.1 Current motivation and mood states

The use of BCIs such as the P300-BCI or SMR-BCI require deliberate engagement of the user with a task for the purpose of active interaction and communication with the system. SMR-BCIs are using motor imagery (MI) paradigms to elicit sensorimotor rhythms (SMR) (SMR-BCIs or MI-BCIs). Similarly frequent are BCI systems for spelling that elicit discrete event-related potentials (ERPs) in the EEG based on the voluntary attention of the user and his/her choice of numbers, letters or symbols rapidly presented in rows and columns. The most traditionally used BCI speller is the well know P300-BCI (for an overview of BCIs, e.g., Edlinger et al., 2015). Consequently, a number of the existing previous studies investigated whether influencing or controlling for the user’s motivation and affective state could intentionally enhance BCI performance in either the P300-BCI or the SMR-BCI. Kleih-Dahms et al. (2021) for example manipulated the user’s mood by experimental mood induction via video clips or music. Examination of the performance of healthy users included SMR-BCI-based motor imagery-induced movement and action control. Moreover, akin to the study by Kleih et al. (2010) that used a P300-BCI, Kleih-Dahms et al. (2021) modulated the user’s motivation experimentally. They set a monetary incentive (extrinsic motivation) and divided the users according to their intrinsic motivation [ratings on visual analog scale (VAS) about their current motivation] into high vs. low motivated users. In a third study by the authors (see Kleih et al., 2011b), data from healthy users and patients diagnosed with amyotrophic lateral sclerosis (ALS) were pooled to further exploit the role of the user’s current motivation for P300-BCI performance. Again, the user’s current motivation was assessed via ratings on the intuitive and easy to use VAS. In addition, the authors used the Questionnaire for Current Motivation (QCM)-BCI to measure motivational dimensions related to BCI use. The QCM-BCI is a standardized questionnaire used in many BCI studies as well (for an overview see Table 1). It is adapted from the QCM (Rheinberg et al., 2001), a questionnaire to assess current motivation in learning situations. The QCM questionnaire is a state measure and allows determining the degree of the achievement motivation of a person (trait) in a situational context (state). Moreover, in a fourth study by Kleih and Kübler (2013), the authors investigated healthy individuals as novices in P300-BCI and divided the users according to their intrinsic motivation (ratings on VAS about their current motivation) or their habitual motivation to help. In addition, a number of further psychological human factors were investigated in the study such as the user’s habitual affective ability to empathize with others (perspective taking), which was measured by self-report using standardized questionnaires (for an overview see Table 1). Furthermore, the user’s memory and ability to pay attention was tested via a cognitive test battery (see Table 1). In some of the previously stated studies that used P300-BCIs, a significant effect of current motivation as measured by self-report scales (VAS) was found (see Table 1). Additionally, the user’s self-reported degree of incompetence fear as assessed with the QCM-BCI was found to correlate with the user’s performance accuracy measures (e.g., spelling accuracy). In the SMR-BCI, a significant effect of reward (experimental manipulation of the user’s current state of extrinsic motivation) was found to influence SMR-BCI performance. The study by Kleih et al. (2010) could not find a significant direct impact of reward on the user’s motivation or BCI performance (spelling) but found differences in P300 modulation during calibration and copy-spelling in the rewarded and unrewarded groups.

The study by Nijboer et al. (2008) investigated the influence of mood and motivation on BCI performance in young adults in the SMR-BCI (see Table 1). In this study, the authors explored the impact of mood and motivation across sensory modalities using auditory or visual feedback during training. The authors used the QCM-BCI to determine motivational facets related to BCI use and assessed changes in the current mood or wellbeing by standardized scales for quality of life (QoL) (for details, see Table 1). The participants performed a motor imagery task to elicit sensorimotor rhythms in the EEG for BCI based control of the participants’ choices: the participants had to select by instruction to play sounds of different music instruments and the choices were rewarded either auditorily (sound related to a selected instrument) or visually by verbal prompts (correct vs. incorrect). During visual feedback, BCI performance varied with the self-reported differences in mood of the healthy users and with their self-reported motivation to use a BCI, in particular with the degree of mastery confidence and incompetence fear reported by the participants in the QCM-BCI. Higher scores of mood and mastery confidence were related to better performance, whereas incompetence fear showed a negative correlation with performance. During auditory feedback, incompetence fear however showed a positive relationship with BCI performance and was the only factor with a significant effect on BCI performance. To elucidate these observations further, in a second study (see Table 1), Nijboer et al. (2010) compared the influence of individual differences in mood and wellbeing and differences in the current motivation of the users on BCI performance among healthy users and patients diagnosed with ALS. The comparison included the performance in two different BCI set-ups comprising SMR- and P300-BCIs. Again, mood and wellbeing were assessed via self-report measures, included scales for QoL as in the previous study, and additionally scales for assessment of depressive symptoms as potential further interindividual modifier of BCI performance (Nijboer et al., 2010). In this study, the authors found a relationship between mood, motivation, and the number of BCI sessions. Moreover, the authors could validate a relationship between BCI performance and motivational factors in half of their participant sample. This included the user’s self-reported challenge, mastery confidence and incompetence fear as measured with the Questionnaire for Current Motivation (QCM-BCI). When comparing the performance of healthy and disabled users, Nijboer et al. (2010) found that motivation for BCI use such as mastery confidence or incompetence fear correlated with BCI performance positively or negatively among healthy or individual ALS users in both, the P300-BCI or the SMR-BCI (Nijboer et al., 2010). However, they could not find a relationship between mood and BCI performance in any of the participants: the scores obtained from the measures of current mood related to QoL and wellbeing or the self-reported current symptoms of depression as further measures of a user’s mood state or trait showed no significant effects.

The findings of the above reported studies suggest that a proportion of the variation in BCI performance is accounted by the user’s motivation among both, healthy users or patients. However with different results across the studies and the measures used, i.e., intrinsic vs. extrinsic motivation, measured experimentally vs. by self-report, and whether related to the performance in the P300-BCI or SMR-BCI, respectively. So far, extrinsic motivation (reward and incentives), the user’s self-reported motivation, as assessed with an intuitive self-report scale (VAS) or as measured in relation to BCI use were often but not consistently correlated with BCI performance across the studies. Moreover, as stated above and as summarized in Table 1, some studies were not able to find any significant effects.

However, as shown in Table 1, some further studies support the suggestion that the motivation of the user plays a role and can affect BCI performance, both in the P300-BCI or the SMR-BCI at least among healthy users. Baykara et al. (2016) used a P300 multi-class speller paradigm. As in the studies discussed above, the authors assessed current motivation in healthy participants with the QCM-BCI and a VAS, akin to the studies by Kleih et al. or Nijboer et al. that were using both or one of these measures. In Baykara et al. (2016), both measures of current motivation showed correlations with BCI performance efficiency assessed via information transfer rate (ITR) and with the amplitude of the P300. Moreover, the participants with high motivation as assessed with the intuitive self-report measure of the VAS had higher P300 amplitudes and higher ITR than the group of participants reporting low current motivation on the VAS (Baykara et al., 2016). The results of this study complement observations from another study conducted by Ahn et al. (2018). This study used the user’s predictions of their BCI performance as a self-report measure of a user’s motivation in the SMR-BCI. In Ahn et al. (2018), healthy participants performed a motor imagery task for SMR-BCI. Between the training sessions, the participants self-reported their current mood, calmness, fatigue, and their mental and physical state were assessed (see Table 1 for details). In addition, the participants should make accuracy predictions about their BCI performance and difficulty of motor imagery. The authors found that the predicted motor imagery performance of the individual user showed a relationship with offline analysis of the participants’ BCI accuracy measures and with the factually achieved classification accuracy.

To conclude, the studies on mood and motivation outlined above and in Table 1 point to the possibility that motivational aspects associated with the use of BCIs impact BCI performance. The motivational dimensions of mastery competence and incompetence fear, the users’ own intuitive evaluation of their task performance (measured by self-prediction or with simple intuitive scales such as the VAS) turn out to be correlates of BCI performance across BCI systems and user groups. Moreover, some of the studies found that in some users, mood may change across sessions either enhancing or decreasing motivation across sessions. However, as discussed in detail in the following sections, as far as mood and affect are concerned, trait variables such as the user’s degree of empathy (e.g., Kleih and Kübler, 2013) or emotional stability as a personality trait (e.g., Hammer et al., 2012) might play a more important role among healthy users than state measures of mood and affect.

### 4.2 Mental states (perception, attention, and cognition)

In an attempt to find best predictors of the user’s traits and states for BCI performance in the P300-BCI or SMR-BCI, a series of previous studies further elaborated and extended the investigation of user variables from motivation and affect or mood to mental states including perceptual and cognitive states of the user. The assessment methods of these studies cover a broad range of perceptual, cognitive, affective or motivational traits and states of the users. Hammer et al. (2012, 2014, 2018) for example used a mixed-method approach that comprised structured psychological test batteries as well as self-report measures to capture a broad range of traits and states (for a detailed overview, see Table 1). The test battery relevant for SMR-BCI performance (Hammer et al., 2012, 2014) included for example perceptual and cognitive indices, sensorimotor coordination, attention, concentration (ability to concentrate on the task and sustain attention to it), or measures of the user’s verbal and non-verbal learning ability. These factors according to the authors critically influenced cognitive performance in a number of tasks in previous research including motor imagery. In addition, to replicate results from earlier studies outlined in section “4.1 Current motivation and mood states,” the authors assessed affective, motivational, and personality variables. These comprised the user’s current mood and motivation, locus of control, Big Five personality traits and clinically relevant current mood symptoms related to anxiety and depressive disorders assessed via standardized self-report measures (for details and comparison with the studies discussed in section “4.1 Current motivation and mood states,” see Table 1). Effects were examined among naive healthy BCI users in the Berlin Brain–Computer Interface (BBCI; Blankertz et al., 2006). The BBCI is based on neurofeedback training and uses direct reinforcement of task-related and task-contingent brain signals of the user. Feedback learning is well known to be an integral part of neurofeedback studies, though not the only constituent to perform well in BCI settings.

From all psychological variables measured, the ability to concentrate on the task and sustain attention to it as well as the user’s sensorimotor skills were significant predictors of the user’s SMR-BCI performance in Hammer et al. (2012). In Hammer et al. (2014), the authors aimed to replicate their results (see Table 1). This revealed two user characteristics, i.e., the user’s visuo-motor coordination ability and the ability to concentrate on the task as significant predictors of SMR-BCI performance. Sprague et al. (2016) also used psychological measures (e.g., current motivation, mood, or fatigue) and psychological tests of cognition that additionally included working memory tasks and on a trait level, the user’s general intelligence. The authors investigated if these factors could be predictors not only of SMR-BCI performance but as well of BCI performance in the P300-BCI among healthy users. The authors found that in P300-BCI, the BCI user’s performance correlated significantly with their performance obtained from the memory tasks. Moreover, general intelligence significantly predicted BCI performance. In addition, the user’s working memory could predict BCI performance but failed as a predictor after adding the additional user traits and states (e.g., the user’s level of fatigue, mood, or motivation). This shows that psychological characteristics of the user, could additionally play a significant role as mediators of BCI performance. Hammer et al. (2018) further exploited the impact of psychological user traits and states on P300-BCI performance. Like in their earlier studies and in the study by Sprague et al. (2016), Hammer et al. (2018) were using an extended psychological test battery that additionally included tests of selective attention that are relevant for attention selection when target stimuli are presented rapidly such as in the P300-BCI spellers (for an overview see Table 1).

The study by Hammer et al. (2018) could not replicate the findings reported in the earlier studies of the authors nor the results reported by Sprague et al. (2016) outlined above. None of the investigated user state factors showed a significant correlation with P300-BCI performance indices in the sample of healthy BCI novices, neither in an auditory nor in a visual version of the P300 speller.

Interestingly, Jeunet et al. (2015) using SMR-BCI investigated the same broad range of psychological self-report variables like for example in the studies conducted by Hammer et al. Additionally, the authors used a broad range of neurophysiological indicators such as changes in EEG frequency bands as predictors of SMR-BCI performance. In line with Hammer et al. (2012, 2014), but in contrast to the results observed in Hammer et al. (2018), Jeunet et al. (2015) found a relationship between a number of cognitive factors and BCI performance in the motor imagery SMR-BCI. Among the sample of healthy users, the ability for spatial mental rotation as well as the user’s habitual level of self-reported tension (impatience and frustration) and the user’s self-reported self-reliance (i.e., the user’s ability to learn autonomously) correlated significantly with the user’s BCI performance across six different training sessions. Besides this, further predictors such as the user’s visuo-spatial memory span or visual or verbal learning abilities contributed to the user’s motor imagery based SMR-BCI performance. However, Jeunet et al. (2015) additionally report that these user characteristics did not predict the user’s BCI performance across all the different training sessions. The training sessions comprised different motor imagery tasks such as left-hand motor imagery, mental rotation or mental subtraction.

Recent studies aimed to scrutinize the potential impact of the user’s cognitive state and cognitive skills on BCI performance, such as the study by Leeuwis et al. (2021). The authors used similar self-report methods as the previous studies outlined in this section but not identical psychological tests and focused on SMR-BCI like most of the afore mentioned previous studies (see Table 1). Like Hammer et al. (2018) for P300-BCI, the study by Leeuwis et al. (2021) could only partly replicate the observations from previous SMR-BCI studies outlined above although investigating a much larger sample size of healthy participants than the sample size of some of the previous studies (see Table 1).

Taken together, the results of the studies summarized in this section underscore the relevance of monitoring the mental cognitive states of the user to ensure accurate BCI performance. However, they also show that cognitive state assessment requires to include a broad number of cognitive states to identify which of the many cognitive variables and cognitive abilities of the user might specifically modulate BCI performance in a given setting within and across the different BCI systems and training sessions.

### 4.3 Role of assessment methods

Therefore, a considerable number of studies have investigated which measures (e.g., neurophysiological, experimental, or self-report) might best predict BCI performance due to the various ways in which cognitive, affective, or motivational states of the user can be measured and how they might influence performance measures in the BCI setting. Of these studies (for an overview see Table 1), a number of studies used external validation techniques to further define the relationship between various measures of a user’s characteristics and states and BCI performance (see Ahn and Jun, 2015 for a brief overview).

For example, Halder et al. (2011) compared the brain activity patterns acquired outside of the BCI context to the performance outcome measures of healthy BCI users obtained from motor imagery driven SMR-BCI (see Table 1). In the study, the same subjects who performed the BCI setting were invited to a second study in which they were asked to perform different motor tasks (e.g., motor observation vs. motor imagery) while their brain activity was monitored by functional magnetic resonance imaging methodology (fMRI) (Halder et al., 2011). The authors grouped the BCI users into those with poor and good BCI performance measures. The authors found that what differentiated best between “poor” and “good” users was the users’ brain patterns in the fMRI experiment and thus there “implicit” ability “to recruit the relevant areas for symbolic motor tasks required for motor imagery and motor observation” (cited from Halder et al., 2011).

In line with this, a series of studies explored the degree to which neurophysiological measures as correlates of a user’s mental state can serve as predictors of BCI performance. Some studies explored the mental state and its neurophysiological correlates during a resting state (e.g., Blankertz et al., 2010). Other studies explored if task-induced changes in neurophysiological activity as biological markers of the user’s cognitive or motor performance can serve as predictors of BCI performance (e.g., Halder et al., 2013; for SMR-BCI: Ahn et al., 2013; Robinson et al., 2018; Tzdaka et al., 2020). Further studies assessed the impact of particular training interventions such as for example mindfulness training or relaxation training on BCI performance (Botrel et al., 2017; Botrel and Kübler, 2019) and for example examined if mindfulness or relaxation training before the start of a BCI session improves motor imagery performance in the SMR-BCI (see Table 1)).

Similar to the studies using psychological measures such as questionnaires or test scores, the results of these studies suggest that many neurophysiological measures are task dependent, BCI type, or performance specific (for an overview see Table 1). In addition, interventions such as short-term relaxation training or mindfulness training, visuomotor coordination training or progressive muscle relaxation seem to have no effects on SMR-BCI performance of healthy BCI users (Botrel et al., 2017; Botrel and Kübler, 2019). A study by Jiang et al. (2021) however found that participants with long-term training in meditation had more stable resting EEG mu rhythms, better resting state SMR predictors than non-mediators, achieved larger control signal contrasts in the motor imagery tasks and fewer subjects in the mediating group were BCI illiterates. For short-term and long-term effects of meditation training interventions on BCI performance testing of healthy users see Tan et al. (2014) or Tan et al. (2015). These latter results corroborate the finding that neurophysiological correlates obtained from the analysis of a user’s mental state during rest (e.g., Blankertz et al., 2010) are reliable predictors of BCI performance. Out of all the neurophysiological predictors investigated thus far, the assessment of activity changes during a resting state appears to predict the user’s unique BCI performance the best in addition to psychological predictors (e.g., Lee et al., 2020). The fact that a resting state increases self-referential processing could be one explanation for these findings (see section “6 Self-relevance as potential superordinate human factor in the BCI setting”).

### 4.4 User traits and BCI performance: the role of personality traits

While several studies examined the impact of user states (e.g., current motivation or mood states or mental states) on BCI performance (see sections above), a considerable number of studies additionally aimed to determine the role of user traits as intraindividually stable differences between subjects as predictors of BCI performance. Most of these studies focused on personality traits. Theoretically, individuals can be distinguished according to a set of fundamental personality traits that predict interindividual variations in human experience and behavior across situations. Thus, personality traits imply stability and may therefore predict a user’s BCI performance across situations. Usually, personality traits are assessed by standardized questionnaires. Most of these questionnaires are based on personality taxonomies and models of personality such as the Big Five-Factor Model (e.g., De Raad and Mlacic, 2015) that distinguishes between Openness, Conscientiousness, Extraversion, Agreeableness, and Neuroticism, respectively. Each of these traits is assumed to be additionally associated with facets of these traits (e.g., extraversion being linked to warmness, assertiveness, positive emotionality, etc. or neuroticism to anxiety, depression, anger, vulnerability, self-consciousness, or impulsivity). As summarized in Table 1, personality traits or facets thereof correlate with BCI performance either positively or negatively in several studies. For example, the studies by Hammer et al. found that “emotional stability” predicted P300-BCI performance among healthy users across the visual and auditory spelling modalities. “Emotional stability” is one of six personality factors from the Big Five Plus One Personality-Inventory used by Hammer et al. (2014) and is associated with the Big Five trait of neuroticism. In the study by Kleih and Kübler (2013), P300-BCI performance correlated negatively with empathy in healthy BCI users (all novices of BCI). Based on these findings, one may speculate that users who are scoring high in empathy and perspective taking might have lower BCI performance, possibly despite or due to their higher motivation to help or their greater incompetence fear compared to user’s scoring lower on empathy and perspective taking.

A study by Bobrova et al. (2018) also discovered this. The authors measured personality traits via standardized questionnaires, and investigated if interindividual differences in neuroticisms - as a personality trait that is related to empathy and emotional stability - modulates SMR-BCI performance. Neuroticism correlated negatively with motor imagery skills and SMR-BCI performance much like Hammer et al. found for emotional stability or Kleih and Kübler (2013) for empathy (see Table 1). The study by Jeunet et al. (2015) that investigated a range of psychological human factors during SMR-BCI (psychological, neuropsychological, or cognitive) and that additionally included the assessment of the user’s personality traits point into a similar direction. In their study, the authors used a version of the Sixteen Personality Factor Questionnaire (16PF, see Table 1). They discovered that during the SMR training sessions, 3 of the 16 personality factors and a fourth factor associated with the users’ reflective and active learning style (tension, abstractness, self-reliance, and self-reliability) accounted for over 80% of the variance in SMR-BCI performance.

Furthermore, the study by Leeuwis et al. (2021) found a significant influence of personality traits on SMR-BCI performance among healthy users. As described in section “4.2 Mental states (perception, attention, and cognition).” Leeuwis et al. (2021) investigated a number of different motivational and cognitive states as well. The authors used the Five Factor Personality Inventory (FFPI) to measure basic personality traits. The five personality factors comprise the personality traits mildness, emotional stability, orderliness, extraversion, and autonomy. BCI performance was accurately predicted by the personality traits “autonomy” and “orderliness” as well as by the vividness of mental imagery across BCI runs. Moreover, the sociodemographic variable “gender” (women performing better than men) affected BCI performance. However, emotional stability did not predict BCI performance across runs as found and reported in other studies (see Table 1). The study conducted by Benaroch et al. (2019) also supports this conclusion. Although personality traits are considered stable and situation-independent, Benaroch et al. (2019) found no effects. The authors used a machine learning approach and data from three different SMR-BCI experiments all using the same study protocol. Personality traits did not predict SMR-BCI performance between different motor imagery tasks in the SMR-BCI across BCI sessions (see Table 1).

Hence, taken together there are differing effects of personality traits on BCI performance. This stands in opposition to studies that found that user’s traits can modulate the user’s BCI experience (see section “2.1 Human factors related to BCI engineering and ergonomics”). In this case, both past and current research suggest a strong correlation between the user’s self-reported operation performance and their self-reported performance satisfaction with their BCI performance (see for a discussion Kübler et al., 2015). In addition, the user’s self-reported and perceived effectiveness (Kübler et al., 2014) and their acceptability of the BCIs as well as their affinity with technology (e.g., Hagedorn et al., 2021; Leeuwis et al., 2021) seem to modulate BCI performance among different vulnerable end-user groups. This may include patients with stroke or with mental health conditions (e.g., Al-Taleb et al., 2019; Voinea et al., 2019). Several of these studies reported effects of these user characteristics on BCI performance measures such as P300 spelling frequency or motor imagery and in an off-line analysis with the brain signals under investigation (e.g., P300 amplitude elicited in a BCI-P300 speller).

## 5 Challenges for future BCI research: finding superordinate key human factors

In summary, the studies described above show that several user traits and states significantly modulate BCI performance in the most well established BCI systems (P300-BCI and SMR-BCIs). The effort of assessing all of these user traits and states with standardized test assessment batteries and different measures however is huge, as pointed out by for example Hammer et al. (2018). Moreover, extended assessment and testing may increase the mental workload or fatigue of the users, and both, workload and fatigue can significantly affect BCI performance negatively as for example reported by Käthner et al. (2014).

Thus, a significant future challenge for user-centered or personalized BCI approaches is the timely or time-efficient assessment of the user’s traits and states. Finding human key factors that influence information processing related to BCI performance across various user groups or BCI systems is one way to address this challenge. Finding superordinate human factors allows discussing how these factors are associated with the user traits and states previously investigated (see Figure 2) and as illustrated in Figure 2 how to implement them as core dimensions in BCI applications for healthy and vulnerable BCI users. Additionally, as illustrated in Figure 4, this may enable the development and proposal of models and study protocols for their standardized assessments across studies (e.g., for designing human factor models; e.g., Jeunet et al., 2017).

Taking into account the results of the studies mentioned above, the following sections will show that the user’s self-concept and the tasks’ and stimuli’s self-relevance may be fundamental superordinate factors of theoretical and empirical relevance for BCI research (for illustrations, see Figures 2–4).

## 6 Self-relevance and the user’s self-concept as potential superordinate human factors in the BCI setting

As a theoretical construct, self-relevance encompasses a number of the affective, cognitive, and motivational human factors examined in earlier BCI studies. Moreover, theoretically and neuroscientifically, self-relevance is a fundamental dimension of information processing (Schmitz and Johnson, 2007). All incoming sensory information is spontaneously appraised according to its relevance for the perceiver. This self-relevant processing is performed during a cascade of sequential appraisal checks including self-referential processing (Scherer et al., 2001; Schmitz and Johnson, 2007). Thus, by inducing self-referential processing, self-relevance determines which stimulus input and information is capturing the user’s attention and which input will be further processed, elaborated, consolidated, and more easily recalled from memory in subsequent sessions and stimulus exposures (e.g., Conway, 2005 or Scherer, 2013). Furthermore, the degree to which an individual’s intentions, behaviors, and self-relevant goals are pursued with effort, ambition, or pleasure is determined by the level of self-relevance induced by tasks or stimuli (Conway, 2005). Therefore, self-relevance can have positive effects on cognition, on emotion and mood and on performance (see Cunningham and Turk, 2017 for an overview).

Thus, enhancing the self-relevance of the stimuli, trials, instructions, and tasks in BCI may improve the users’ performance accuracy across different BCI systems. This can include passive as well as active or reactive BCI systems such as the P300-BCI or the SMR-BCI, respectively.

### 6.1 Enhancing self-relevance via stimulus-driven or top-down triggered processing

Several strategies might increase the self-relevance of a BCI setting (see Figure 2 for an example). One way of increasing self-relevance is through stimulus-driven processing that is bottom-up and initiates implicit, self-relevant and self-referential appraisal processes (Schmitz and Johnson, 2007). For example, the presentation of self-referential stimuli, like the subject’s own name, can initiate self-referential processing and modulate the P300 amplitude (e.g., Tacikowski et al., 2011; for reviews see e.g., Knyazev, 2013) relevant in many BCI studies for classification. Self-referential stimuli such as the subject’s own name spontaneously guide the user’s attention, motivation or action without requiring the user’s explicit attention or intention. On the other hand, explicit task-instructed processing that is top-down driven can enhance self-relevance (e.g., Schmitz and Johnson, 2007). Top-down driven explicit self-relevant processing can be induced via task instructions that promote self-referential processing, e.g., by asking participants to process the task and stimuli from a first person perspective. First-person processing evokes a sense of agency and ownership (for an overview see e.g., Northoff et al., 2006; Schmitz and Johnson, 2007) thereby increasing self-relevance. By using self-referential stimuli or self-referential task instructions, self-relevance increases because BCI users can relate stimuli, objects, feelings, thoughts, and actions to themselves and experience themselves as agents and owners of the task and actions. In contrast to stimulus-driven processing, task-instructed self-referential processing requires introspection (e.g., see Northoff et al., 2006; Northoff, 2016). Therefore, inducing self-relevance by stimulus-driven self-referential processing could be a strategy to improve BCI performance even among severely impaired end-users of a BCI (see e.g., Magliacano et al., 2019 and section “6.2 Implementing self-relevance in the BCI setting: first evidence from P300-BCI and SMR-BCI and future examples”). On the contrary, inducing self-relevance by task instructed self-referential processing requires a BCI user who can reflect upon the self as an owner of experience (e.g., see Northoff et al., 2006; Northoff, 2016).

### 6.2 Implementing self-relevance in the BCI setting: first evidence from P300-BCI and SMR-BCI and future examples

Some previous BCI studies have already made use of the benefits of self-relevance (for an overview see Figure 3). For example, Neuper et al. (2009) used a SMR-BCI in which some sessions comprised self-relevant (feedback with a grasping hand) vs. abstract feedback (moving bar). BCI performance did not differ between sessions in these studies. Therefore, to increase the self-relevance of the BCI setting for the user, recent BCI studies have replaced the flanker stimuli (indicating left or right hand movements) in the mental imagery training conditions in SMR-BCIs by self-referential stimuli such as the user’s own virtual arm (e.g., Škola and Liarokapis, 2018; Ziadeh et al., 2021). The results of these studies found that healthy young adults performed the SMR-BCI equally well in both conditions (own arm vs. flankers). Furthermore, replacing the flankers by the virtual arm in the study by Ziadeh et al. (2021) elicited sense of agency and sense of ownership among the users. This, according to the authors, prevented loss of motivation across 30 trials of repeated motor activation by mental imagery. Likewise, the study by Škola and Liarokapis (2018) found no decline in accuracy measures during the different sessions. Moreover, the participants’ sense of ownership induced during the self-relevant conditions correlated with the modulation of the sensorimotor rhythm used for BCI classification. Stimulus-driven self-relevant processing has been included in P300-BCIs as well. A recent study by Lu et al. (2020) presented self-referential cues and primes such as the user’s own vs. others faces or names during spelling to increase the self-relevance of the spelling sessions and spelling accuracy. Yet another study by Dong et al. (2015) asked healthy BCI users to read self-relevant sentences to increase self-relevance. The classification accuracy of the BCI algorithms (using time-frequency measures of the EEG) improved significantly. A BCI classification accuracy of 75.5% was obtained. This allowed successful discrimination between two different implicit intentions of the user related to agreement or disagreement (Dong et al., 2015).

Taken together and as summarized in Figure 3, as far as stimulus-driven self-relevant processing is concerned, most previous BCI research have focused on increasing self-relevance by triggering self-referential processing implicitly by means of self-referential and highly salient and familiar stimuli such as the subjects own name, own face, voice, or own body parts. This included studies with severely impaired patient groups such as patients with disorders of consciousness as potential user groups of BCIs (e.g., Laureys et al., 2007; Perrin et al., 2015; Hammer et al., 2018; Kempny et al., 2018; for an overview, see Magliacano et al., 2019).

Stimulus-induced self-relevant tasks, that have so far not been used in a BCI setting but seem promising, are tasks that use visual or auditory self-referential vs. other-referential first person vs. third person stimuli, such as personal or possessive pronouns (e.g., Blume and Herbert, 2014; Herbert et al., 2016; Zhou et al., 2019). Due to their simplicity, these paradigms could be a means of facilitating implicit self-referential processing during the BCI training sessions to improve or avoid loss of agency and ownership during spelling or motor imagery. Moreover, promoting self-referential processing of the BCI user, for example, with self-referential instructions or self-referential primes (e.g., personal pronouns or the user’s own name) could be of particular relevance when sense of agency is severely compromised. This can be the case, when controlling a robotic or virtual arm, hand or limb via BCIs (e.g., Caspar et al., 2021; for a discussion of agency in SMR-BCI, see Jeunet et al., 2016).

A number of previous BCI studies explicitly instructed the users to initiate top-down driven self-relevant processing. For example, self-referential instructions that ask participants to imagine actions during motor imagery from a self-referential first person perspective have proven superior for BCI performance in the SMR-BCI over non-self-referential instruction or imagination from a self-detached third person perspective (e.g., Neuper et al., 2005). In line with these results, Placidi et al. (2018) proposed a BCI user interface in which the task is to evoke emotions by instructing the users to imagine self-induced autobiographical events with affective impact. Once the different emotions are detected, the self-referential processing task could be used to train the classifiers to improve communication in severely disabled users with language and cognitive deficits (Placidi et al., 2018).

Strong support that self-relevance – implicit, stimulus-driven or explicit and top-down controlled – plays a superordinate role in BCI designs comes from studies that included a resting state recording. As discussed in section “4.3 Role of assessment methods,” indicators derived from the so-called resting state paradigm have proven one of the strongest predictors for high performance accuracy in SMR-BCIs. Previous BCI studies revealed that there exists a relationship between the resting-state brain activity and SMR-BCI performance (Blankertz et al., 2010; Zhang et al., 2015; Kwon et al., 2020). In line with this, individual differences in the measures of SMR in the resting state (rs-SMR) have been found to predict control over SMR power during the SMR training sessions (e.g., Reichert et al., 2015). In the so-called resting state paradigm, the participants are instructed to relax with eyes open or eyes closed to let their mind wander. Research using these task settings (resting state with eyes open/closed) have shown that the resting state evokes significant alterations in the activity of the brain’s attention-, default-, and salience networks. This includes cortical midline structures (CMS); i.e., brain structures and networks that change activity during self-referential processing (Northoff et al., 2006; Sheline et al., 2009; Costumero et al., 2020). Moreover, the resting state modulates activity in the sensorimotor network (SMN) (Biswal et al., 1995) that comprises the supplementary motor area and the primary motor cortex engaged in the execution of voluntary movements. In addition, resting state conditions facilitate salient stimulus processing, which improves cognitive performance, attention, and memory processes (Kober et al., 2015). Moreover, EEG-neurofeedback training based on oscillations stemming from CMS structures that modulate self-referential processing already proved to be a promising tool to enhance self-regulation abilities in patients with ALS in the late stage of the disease (e.g., Fomina et al., 2016).

These results could explain why a resting state predicts the outcome measures in SMR-BCIs in previous studies. The results suggest that a resting state is a state of increased self-referential processing that modifies neurophysiological activity in specific brain networks of particular relevance for BCI applications such as SMR-BCI. Many recent studies meanwhile incorporate a resting state measure in the BCI training in their study guidelines. These studies include designs with motor imagery and SMR-BCI (for recent suggestions see Zhang et al., 2020) or with P300 spelling (e.g., Won et al., 2022).

### 6.3 Assessing the self of the user: self-concept, its relationship to user traits examined in previous BCI studies and its relevance for future BCI studies

Self-relevance is a theoretical concept closely related to the self of a person and the person’s self-concept. However, self-relevance characterizes a processing state of information (see section “6.1 Enhancing self-relevance via stimulus-driven or top-down triggered processing” for details) that changes in accordance with the stimuli, task, instruction, or the circumstances at hand (e.g., the user’s mood, motivation, or behaviorally self-relevant goals pertinent to the self). In contrast to self-relevance, the self-concept is related to the concept of who we are including a person’s self and identity. Theoretically, the self-concept is multidimensional, comprises multiple facets and encompasses all processes, ideas, attitudes, and beliefs a person may form about the own identity, abilities, experiences, and behaviors. This includes beliefs about personality traits, physical characteristics and affective and cognitive self-judgments (Baumeister, 1999; McLeod, 2008). Therefore, a person’s self-concept can be theoretically considered as a superordinate human factor that predicts a person’s behavior across situations similar to personality traits. There exist several questionnaires that measure a person’s self-concept and that assess self-relevance and self-referentiality on the individual level as a habit or trait. The Personal Self-Concept Questionnaire (PSQ; Goñi et al., 2011) for example assesses a person’s self-referentiality on the dimensions of self-fulfillment, autonomy, honesty, and emotionality (emotional self-concept). Other questionnaires include self-esteem questionnaires such as the Rosenberg self-esteem scale (Rosenberg, 1965). This scale assesses the degree of the person’s self-confidence vs. self-depreciation. In addition, implicit self-report measures for the assessment of a person’s self-concept exist. The Twenty-Statement Test (TST; Watkins et al., 1997) for example allows cross-cultural assessment of self-attitudes. In the TST participants are asked to answer the question “Who am I.” A modified short version of the TST has recently been successfully used in a survey study in combination with machine learning methods to investigate changes in the self-concept and the mental state and academic performance among healthy adults (students) during the COVID-19 pandemic (Herbert et al., 2021).

Recent research, following previous studies asking BCI users about their experiences using a BCI, showed that many BCI users value achieving independence, autonomy, and social participation by using BCIs. Of additional relevance for the BCI users are the experience of happiness or joy and creativity as well as the consideration of BCIs as a means of self-expression (for a summary and overview see e.g., Kögel et al., 2020). Moreover, several previous BCI studies discussed in the previous sections of this manuscript have investigated facets of the BCI user’s personality. This has shown that scoring high or low on measures of current motivation or on personality traits related to empathy or emotional stability and neuroticism can affect BCI performance across BCIs positively or negatively. Differences in a person’s self-concept and self-esteem have been discussed in the literature as predictors of competence, cognitive performance, happiness, interpersonal success, and even of healthier lifestyles (for a discussion, see e.g., Baumeister et al., 2003). Therefore, it is likely that the user’s self-concept modulates the autonomy and happiness achievement goals of a BCI user that have influenced BCI performance in previous BCI studies (see section “4 Psychological human factors and their impact on BCI performance: evidence from previous studies using P300-BCI or SMR-BCI among healthy and disabled user groups” and Table 1 for a detailed summary).

For the reasons stated above, it would be interesting to examine the relationships between the self-concept of BCI users and personality traits used in previous BCI studies, including the users’ beliefs about their performance competencies. Furthermore, self-concept scales and self-esteem scales can be a reliable way to measure the BCI users’ beliefs about and confidence in their own abilities. This can be especially useful in adjusting for overestimation and underestimation biases in self-enhancement motivation and cognitive performance (Alicke and Sedikides, 2009). Identifying whether BCI users overestimate or underestimate their BCI performance could support giving accurate performance feedback during BCI training sessions (for a discussion of positive feedback, see e.g., Lotte et al., 2018). This could help prevent user over- or underestimation biases as well as loss of motivation such as mastery confidence and loss of self-regulation, all of which can eventually decrease BCI efficiency.

Furthermore, previous studies indicate that healthy individuals typically exhibit optimistic biases as well as positively biased affective evaluation of themselves (e.g., Sharot, 2011). These self-positivity biases can be measured experimentally. For example healthy people often display a tendency to prioritize positive over negative trait adjectives when asked to ascribe these to the self (Matlin, 2016). Studies using EEG have also discovered this bias. For instance, when healthy participants are processing rapidly presented emotional adjectives in rapid serial visual presentation designs (Herbert et al., 2008), or read emotional and neutral first vs. third person pronoun-noun pairs (Herbert et al., 2011) or social vignettes that are either self- or other-relevant (Fields and Kuperberg, 2016). In the EEG, self-referential processing of positive linguistic input modulates a number of event-related brain potentials including early and late ERPs. Later ERP modulation includes modulation of the N400 potential during reading of positive vignettes or of late positive potentials (LPP) during processing of self-related positive word and pronoun-noun pairs. The modulation patterns suggest that positive information is better semantically integrated and more deeply elaborated compared to negative or neutral input that is unrelated to the self.

Theoretically, a positive emotional self-concept in terms of positively biased self-views, high self-esteem, high self-positivity, and positive mood have a positive psychological benefit for a person. Self-positivity and positive mood promote cognitive processes by broadening attention, by fostering motivation, self-esteem, and performance (for a discussion, see Fredrickson, 2004). Patients with mental disorders and depressed mood can possess compromised self-views, low self-positivity or negativity biases, and self-esteem. Moreover, self-evaluations can extend from human–human to human–computer interaction (Moon, 2003; Weis and Herbert, 2022).

Therefore, promoting positive self-views in BCI users during the training sessions should help to promote accurate BCI performance, given the negative relationships that have been found in the past between empathy or emotional stability and BCI performance. Integrating self-concept measures in future BCI studies and examining their relationship with the user traits and states investigated in previous BCI studies seems mandatory to monitor the self-serving attributions of the BCI user that could modulate BCI performance positively or negatively. As outlined above and as illustrated in Figures 3, 4, short assessment tools using free-format responses or self-other referential words and vignettes could be a promising and time-efficient tool to determine the BCI user’s self-concept including the users’ self-views and self-attributions in addition to standardized self-evaluation questionnaires.

## 7 Conclusion and recommendations for the future

Previous research targeting the user’s traits and states and investigating their impact on the BCI user’s performance during P300-BCI or SMR-BCIs have provided evidence that a considerable number of user traits and states can influence BCI performance of healthy users and patient users. Based on that evidence, the proposal to search for key human factors seems promising because as explained in detail in the above sections, it could integrate many different user traits and states. Self-relevance could be such a potential key human factor for BCIs in future because as argued in this manuscript, the self-relevance of stimuli and task might influence information processing of the BCI user during multiple stages by increasing attention, agency, and ownership. Moreover, incorporating measures of the user’s self-concept might help promoting positive self-views and avoiding negative self-views of the user about their BCI performance. As outlined in this manuscript, the research on the impact of self-relevance and the self-concept on BCI performance is still in its early stages. This manuscript and the hypothetical model shown in Figure 4 may serve as a catalyst for further research highlighting the potential of these two constructs as important human factors to be thoroughly investigated in future BCI studies with regard to BCI performance in P300-BCIs or SMR-BCIs as well as beyond. From the perspective of BCI applications and BCI engineering, this might support the development of self-referential BCIs for a broad range of users. Due to their self-referentiality, self-referential BCIs could closely fit to the individual user with respect to the user’s needs, traits, and states and improve BCI literacy and BCI accuracy of BCI users beyond the current level. Self-referential BCIs may include the application of self-paced BCIs (e.g., Mason et al., 2006; Scherer et al., 2007) or passive BCIs because these BCIs need to autonomously decide whether the ongoing brain activity is intended by the user (healthy or disabled) or not (self-paced BCIs) and reflecting the user’s traits or states (passive BCI).

## Author contributions

CH: Writing – original draft, Writing – review & editing, Conceptualization, Investigation, Methodology, Validation, Visualization, Formal analysis, Project administration, Resources.
